# Differential influences of unilateral tDCS over the intraparietal cortex on numerical cognition

**DOI:** 10.3389/fnhum.2015.00110

**Published:** 2015-03-05

**Authors:** Christina Artemenko, Korbinian Moeller, Stefan Huber, Elise Klein

**Affiliations:** ^1^LEAD Graduate School, Eberhardt Karls University of TuebingenTuebingen, Germany; ^2^Knowledge Media Research CenterTuebingen, Germany

**Keywords:** transcranial direct current stimulation, unilateral tDCS, number magnitude processing, place-value processing, numerical cognition, intraparietal sulcus

## Abstract

Recent neuro-imaging research identified the bilateral intraparietal sulcus (IPS) to be a key area associated with number processing. However, causal structure-function relationships are hard to evaluate from neuro-imaging techniques such as fMRI. Nevertheless, brain stimulation methods like transcranial direct current stimulation (tDCS) allow for investigating the functional relevance of the IPS for number processing. Following up on a study using bilateral bi-cephalic tDCS over the IPS, the current study aimed at evaluating the differential lateralized functional contributions of the left and right IPS to number processing using unilateral bi-cephalic tDCS over either the left or right IPS. Results indicated a right lateralization for the processing of the place-value structure of the Arabic number system. Importantly, the processing of number magnitude information was not affected by unilateral IPS corroborating the assumption that number magnitude is processed in the bilateral IPS. Taken together, these data suggest that even though number magnitude is represented bilaterally, the left and right IPS seem to contribute differentially to numerical cognition with respect to the processing of specific other aspects of numerical information.

## Introduction

Magnitude information is the most important information conveyed by Arabic numbers (Shepard et al., 1975; Miller and Gelman, 1983; Miller and Stigler, 1987, 1991). On the neural level, there is accumulating evidence from both, patient studies as well as functional neuro-imaging studies that the bilateral intraparietal sulci (IPS) are a core region for number magnitude processing. The IPS is activated in basic numerical tasks such as number magnitude comparison (e.g., Chochon et al., 1999; Le Clec’H et al., 2000; Pinel et al., 2001; Klein et al., 2010a; Wei et al., 2014; for a review, see Dehaene et al., 2003; see also Rugani et al., 2015, for evidence on a spatial representation of number magnitude in chicks), but also in more complex arithmetic procedures such as addition and subtraction (e.g., Kong et al., 2005; for a review, see Arsalidou and Taylor, 2011). For instance, Klein et al. (2009) investigated the neural correlates underlying multi-digit addition. In a two-digit addition task participants had to choose the solution probe from two alternatives, which was either identical with the correct result or closest to it. In particular, the authors manipulated the factors target identity and distractor distance. While target identity specifies whether the target is the actual correct result or the probe closest to it, distractor distance indicates the distance between target and distractor. Additionally, the stimulus set was balanced perfectly regarding a range of control factors including the need for a carry-over (i.e., 29 + 38 vs. 25 + 42, carry-over needed whenever the sum of the units of the operands exceeds 9). In line with recent data on mental arithmetic, influences of target identity were—among others—associated with fMRI signal change in and around the bilateral IPS as were influences of distractor distance and carry-over.

Even though the bilateral intraparietal activation pattern fits nicely into the notion of the bilateral IPS being critically involved in number (magnitude) processing, its functional involvement and, thus, a reliable structure-function relationship cannot be inferred from fMRI data. However, transcranial direct current stimulation (tDCS) as a non-invasive brain stimulation method allows for investigating the functional involvement of cortical sites identified by fMRI in a given task (cf. Brunoni et al., 2012 for a review). By stimulating a specific brain area the neural activity of this target area can be influenced by shifting cortical excitability (Nitsche et al., 2008). Investigating the causal structure-function relationship of the bilateral IPS for number magnitude processing, Klein et al. (2013) conducted a study evaluating the influence of bilateral bi-cephalic tDCS with two active electrodes of the same polarity applied to the IPS on the factors distractor distance, target identity and carry-over in the above described two-digit addition task. In a within-task comparison, results indicated that the influence of distractor distance was moderated by bilateral tDCS stimulation: A reliably smaller distractor distance effect under anodal as compared to cathodal stimulation clearly indicated an influence of tDCS on participants’ ability to process number magnitude. In contrast, the effects of target identity and carry remained unaffected. Moreover, the stimulation effects were specific to number processing as revealed by a between-task comparison: more general cognitive functions were not influenced as indicated by no stimulation effects on a controlling color word stroop task. Therefore, Klein et al. (2013) suggested a functional involvement of the bilateral IPS in number magnitude processing and thus corroborated a core assumption of the Triple Code Model (Dehaene et al., 2003) proposing a bilateral representation of number magnitude information in the human brain.

Importantly, however, Klein et al. (2013) only applied bilateral stimulation without a control for possible unilateral stimulation effects. Thus, the conclusion that the bilateral IPS is functionally involved in number magnitude processing would be further corroborated when unilateral bi-cephalic application of tDCS over the left or right IPS, respectively, would not modulate the distractor distance effect. Additionally, it would be interesting to evaluate whether the effect of target identity, which was not influenced by bilateral stimulation, may be moderated by unilateral stimulation. Therefore, the aim of the current study was to specifically evaluate differential influences of the left and the right IPS to number processing in above described two-digit addition task.

In a recent related study, Hauser et al. (2013) investigated the influence of anodal unilateral tDCS over the IPS on number magnitude comparison as well as two-digit subtraction. Comparing stimulation induced changes to a sham condition the only reliable stimulation effects were a speeding of reaction time (RT) in number magnitude comparison and a reduction of RT in the subtraction task after anodal stimulation over the left IPS. On the one hand, this indicates that unilateral tDCS can influence number processing. On the other hand, however, it also demonstrated that a more systematic investigation of both stimulation sides (left vs. right hemisphere) and types (anodal vs. cathodal stimulation) would be desirable to further evaluate the causal structure-function relationship between the bilateral IPS and specific components of number processing.

Taken together, bilateral IPS activations were observed for distractor distance, target identity, and the need for a carry-over (Klein et al., 2009). However, when stimulating the bilateral IPS with tDC only the distractor distance effect was altered (Klein et al., 2013). This substantiates the causal structure-function relationship of the bilateral IPS for effects drawing directly on the number magnitude representation such as the numerical distance effect. Yet, there is also empirical evidence for a functional lateralization of number processing components suggested by studies on children with dyscalculia (Price et al., 2007), studies applying transcranial magnet stimulation (TMS; Cohen Kadosh et al., 2007, 2012; but see Cappelletti et al., 2009) and lesion studies (Zorzi et al., 2002; but see Pia et al., 2009). On the one hand, Price et al. (2007) observed a reduced activation of the right IPS associated with impaired magnitude processing in dyscalculia. In line with this, Cohen Kadosh et al. (2007, 2012) found similarly impaired magnitude processing induced by TMS over the right but not the left IPS. On the other hand, Cappelletti et al. (2009) reported evidence for a functional involvement of both, the left and the right IPS in number magnitude processing using TMS. Interestingly, however, impairments on number magnitude processing were more pronounced when TMS was applied to the left rather than the right IPS. Furthermore, Zorzi et al. (2002) found a disturbed representation of number magnitude in patients after right-hemispheric brain lesion, whereas Pia et al. (2009) showed it for a single case of left-hemispheric brain lesion. Taken together, this illustrates that the functional role of the right and left IPS in number processing is still to be resolved.

Therefore, the current study set off to appraise the causal structure-function relationship between the left and the right IPS, respectively, and different components of number processing, (i) number magnitude processing as reflected by the distractor distance effect; (ii) recognizing familiarity as represented by the target identity effect; and (iii) place-value processing as indicated by the carry effect, by means of bi-cephalic unilateral tDCS.

In line with the results of Klein et al. (2013) we do not expect the distractor distance effect to be moderated by unilateral tDCS, since number magnitude is considered to be processed in the IPS bilaterally. On the other hand, we hypothesized that tDCS over the right IPS should influence the carry effect because the processing of place-value information has been associated specifically with intraparietal cortical areas in the right brain hemisphere (Whalen and Morelli, 2002; Göbel et al., 2004; Wood et al., 2006). The effect of target identity, however, we expected to be manipulated by tDCS over the left IPS, because—with the target being the correct solution in half of the problems—it may rather reflect processes of familiarity recognition. Together with processes of recollection, processes of familiarity have been associated with fact retrieval (e.g., Montaldi and Mayes, 2010) with its neural correlates in the angular gyrus (Klein et al., 2013) and the retrosplenial cortex (Vann et al., 2009; Montaldi and Mayes, 2010; Klein et al., 2013 for a review).

## Materials and methods

### Participants

Twenty-five student volunteers (22 females; mean age: 23.28 years, *SD* = 4.51 years) provided informed consent and received monetary compensation for successfully completing the study. All participants were right-handed as assessed by the Edinburgh-Handedness Inventory (Oldfield, 1971), native German speakers and showed no history of neurological or psychiatric disorders. The study was approved by the local ethics committee of the Medical Faculty of the Eberhard Karls University of Tuebingen.

### Design

The experimental design of the study was a 2 × 2 × 2 within-subject design discerning the factors stimulation type (i.e., anodal vs. cathodal), stimulation site (i.e., left vs. right hemisphere), and task (i.e., mental addition vs. color word stroop). Additionally, there was also a condition of sham stimulation. Thus, there were five stimulation conditions in total (i.e., right cathodal, right anodal, left cathodal, left anodal, and sham) under which each participant had to perform the addition and a color word stroop task.

The *addition task* was virtually identical to the one employed by Klein et al. (2009). For each of the five stimulation conditions a matched stimulus set of 192 two-digit addition problems was created. Due to limitations in the number of possible addition problems and matching constraints an item was repeated three times at most across but never within stimuli sets. Participants had to decide which one of two solution probes was the correct or closest to the correct sum by pressing a corresponding button. For the addition task, the factors carry-over (needed vs. not needed, e.g., 29 + 38 vs. 25 + 42, i.e., carry over needed whenever the sum of the units of the operands exceeds 9), target identity (whether the target was either the correct sum or the sum closest to it), and distractor distance (small, i.e., 4–9, vs. large, i.e., 14–19, numerical distance between target and distractor, for more details see Klein et al., 2009) were manipulated orthogonally. Stimulus groups and sets were carefully matched regarding different variables including numerical size and parity of the operands, target and distractor distance, distance between target and correct result, distance between distractor and target as well as the overall distance between distractor and correct result, etc.

Addition problems together with two solution probes were presented in white Arabic notation against a black background until either a button was pressed or the time limit of 5000 ms was reached directly followed by a fixation cross presented for 500 ms. Testing was preceded by a training phase of 32 trials to familiarize participants with the arithmetic task and the experimental set-up (cf. Klein et al., 2013). The practice items were not included in the testing phase.

A color word *stroop task* served as a control task in which color words were presented in different colors centered on a black screen. Participants were instructed to identify the color of the presented word and to press a corresponding button on the keyboard. Only in congruent trials the ink color matched the presented color word. Stimuli were presented until a button was pressed or the time limit of 2000 ms was reached. The next trial started with a fixation cross for 300 ms followed by a blank screen for 500 ms. The stroop task consisted of 24 practice trials followed by 96 critical trials with 50% of the critical trials congruent and 50% incongruent. Trial order was randomized.

### Stimulation method

In this study, bi-cephalic tDCS was applied to the left and right IPS, respectively, since target and distractor effects were associated with bilateral activations of the IPS (BA 7 and BA 40) and the posterior IPS (BA 7) (Klein et al., 2009). As described by Klein et al. (2013), these regions correspond to the positions P3 and P4 according to the international 10–20 system for EEG electrode placement (cf. Okamoto et al., 2004), which was additionally validated by Klein et al. (2013) using MRI scans.

**Figure 1:**
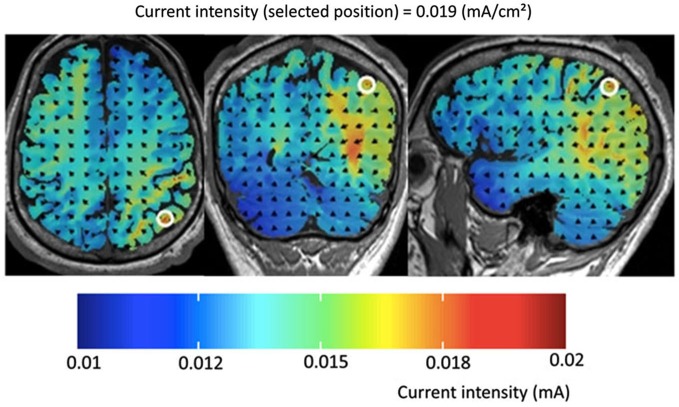
**Results of the computer simulation for the experimental set-up used in the present study for tDCS over P4 (and vice versa for P3)**. Please note that current intensity did not exceed 0.015 mA. Thus, current density was below 0.01 mA/cm^2^ in frontal brain areas but reached values equal or larger than 0.019 mA/cm^2^ in the intraparietal cortex. According to Nitsche and Paulus (2000) a minimum current density of 0.017 mA/cm^2^ is necessary to modify cortical excitability by tDCS in humans.

For each stimulation condition an electrode size of 5 × 5 cm^2^ was used for the two square scalp electrodes at the target positions P3 and P4 (one experimental, one sham) and an electrode size of 10 × 10 cm^2^ for the two reference electrodes placed in the supra-orbital region, whereby all electrodes were covered with conductive rubber and saline-soaked synthetic sponges. As in the study of Klein et al. (2013), a current of 1 mA was applied to the target regions via two independent channels and so the resulting current density was 0.04 mA/cm^2^ for the target electrode and 0.01 mA/cm^2^ (= 1 mA/100 cm^2^) for the inactive reference electrodes, respectively (see Figure 1). According to Nitsche and Paulus (2000) a minimum current density of 0.017 mA/cm^2^ is necessary to modify cortical excitability by tDCS in humans. For the tDCS a multichannel DC Brain Stimulator device (NeuroConn, Illmenau, Germany) was used.

In anodal and cathodal stimulation conditions, direct current was applied constantly for a duration of 20 min. Stimulation was preceded by a ramp-up phase in which the current was slowly increased for 15 s until it reached the stimulation threshold of 1 mA. Towards the end, stimulation was ramped down by decreasing the current 15 s until it turned off. In the sham condition, current was ramped up for 15 s, constantly delivered for 30 s and ramped down again for 15 s.

Unilateral stimulation was applied to either the left or the right IPS, respectively, with the effective electrode placed at either P3 or P4, a sham electrode placed on the contralateral hemisphere (P3 in case of an active P4 electrode). The active (but ineffective) reference electrode was placed in the supra-orbital region contralateral to the stimulating electrode whereas the sham reference electrode was placed ipsilateral. The non-stimulating channel followed the sham procedure to prevent that participants got aware of the unilateral stimulation as well as stimulation side. This way, only one additional sham session was required for providing a baseline for both sides (left/right) and stimulation types (anodal/cathodal).

### Procedure

Each subject participated in five sessions consisting of the different stimulation conditions. Order of stimulation conditions was counterbalanced across participants. Between the sessions there was a minimum interval of 6 days (*M* = 9.67; *SD* = 4.88; *R* = 6–29 days) to avoid short-term training effects and long-term stimulation effects (Cohen Kadosh et al., 2010). During tDCS, participants were sitting approximately 50 cm in front of the screen. The training phase of the addition task started simultaneously with the tDCS application according to the respective stimulation condition. To establish the tDCS effect, the testing phase of the experimental addition task was initialized approximately 5 min after stimulation onset. The control stroop task was conducted immediately after the addition task. Stimulation was terminated after 20 min or when the participant finished the experiment.

### Analysis

Statistical analysis of RTs was performed using R (R Development Core Team, 2012). The analysis involved two steps to first evaluate possible overall effects of stimulation and test our specific hypotheses afterwards. In the first step, we analyzed the effect of stimulation on RT by running a linear mixed effects model (LMM) including the factor stimulation (sham, right anodal, left anodal, right cathodal, and left cathodal) as fixed as well as random effect. This analysis was run both for the critical addition task as well as the color word stroop control task.

In the second step, we evaluated the effect of stimulation type without sham on the carry effect, the distractor distance effect, and the target distance effect. As we had specific hypotheses for the left and right hemisphere, separate LMMs for each stimulation side (right or left hemisphere) were run. We included the interactions of stimulation type (anodal vs. cathodal) with (i) carry (needed vs. not needed), (ii) distractor distance (small vs. large), and (iii) target identity (identical vs. non-identical) as well as the respective main effects as fixed effects. Due to the large number of fixed effects, we only included the respective two-way interaction and main effects as random effects when testing for the significance of a specific interaction (cf. Barr et al., 2013). Additionally, we included a random intercept for participants as well as items in all analyses. Fixed effects in the analysis of the color word stroop task were stimulation type and congruency (congruent or incongruent) as well as their two-way interaction. Again, LMMs were conducted for the left and right hemisphere separately. The random effects structure was kept maximal for the analysis of the color word stroop task including all fixed effects as random effects as well as random intercepts for participants and items.

The *p*-values for the effect of stimulation were obtained using likelihood ratio tests. To obtain *p*-values for the analyses of the effect of stimulation type on the carry-over, distractor distance, target identity and color word stroop effect we used the R package lmerTest (Kuznetsova et al., 2013), which calculates degrees of freedom using the Satterthwaite approximation. Since a standard procedure for calculating effect sizes for LMMs is not yet established, the standardized effect sizes were calculated by z-transforming RTs. All factors were effect coded prior to data analysis to allow for Type III tests of fixed effects.

## Results

### Addition task

In a first step, running the LMM including only the effect of stimulation revealed that the effect of stimulation on RT was not significant indicating that overall RT were not influenced by stimulation (right anodal: 2788 ms, left anodal: 2739 ms, sham: 2722 ms, right cathodal: 2693 ms, left cathodal: 2714 ms; *F*_(4,23.93)_ = 0.38, *p* = 0.819).

**Figure 2:**
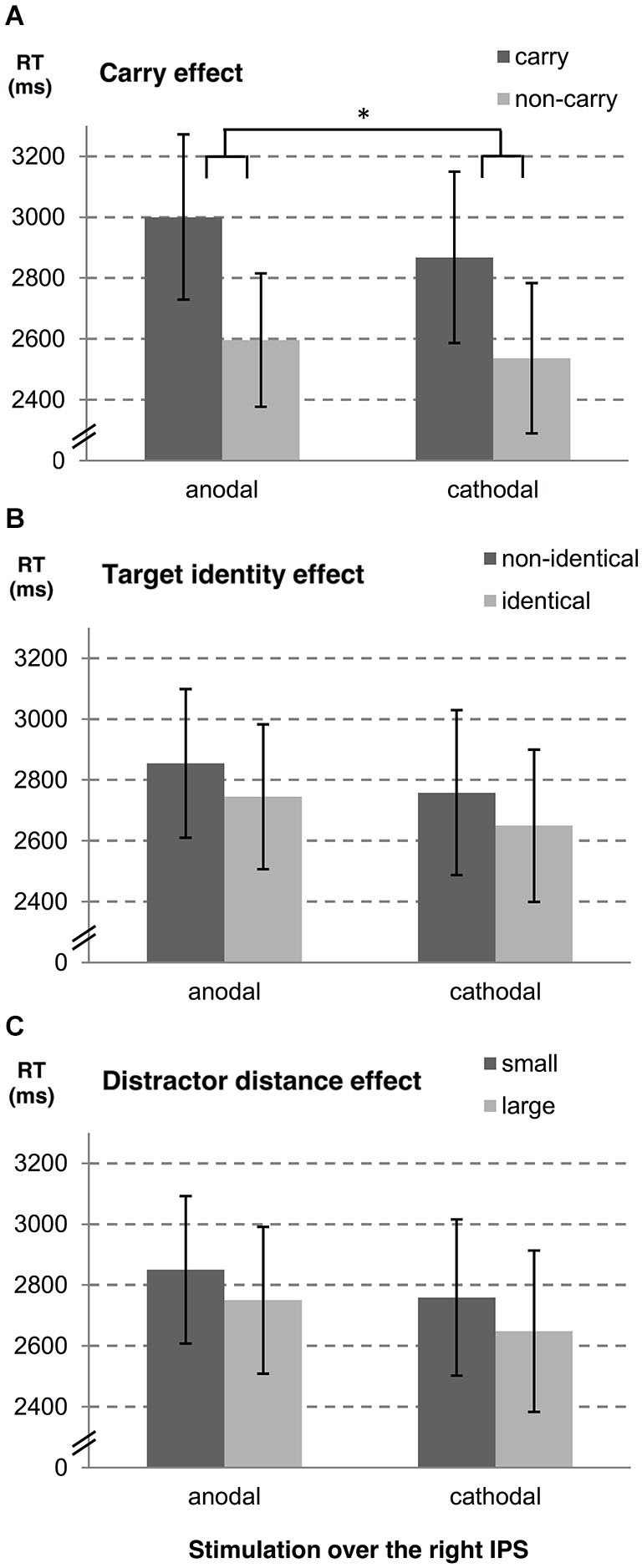
**Influences of tDCS over the right IPS on arithmetic performance**. The stimulation effects for the right hemisphere are shown for **(A)** carry-over, **(B)** target identity and **(C)** distractor distance. Note that the stimulation modulated the carry effect significantly (as indicated by “*”). Error bars indicate the coincidence intervals.

**Figure 3:**
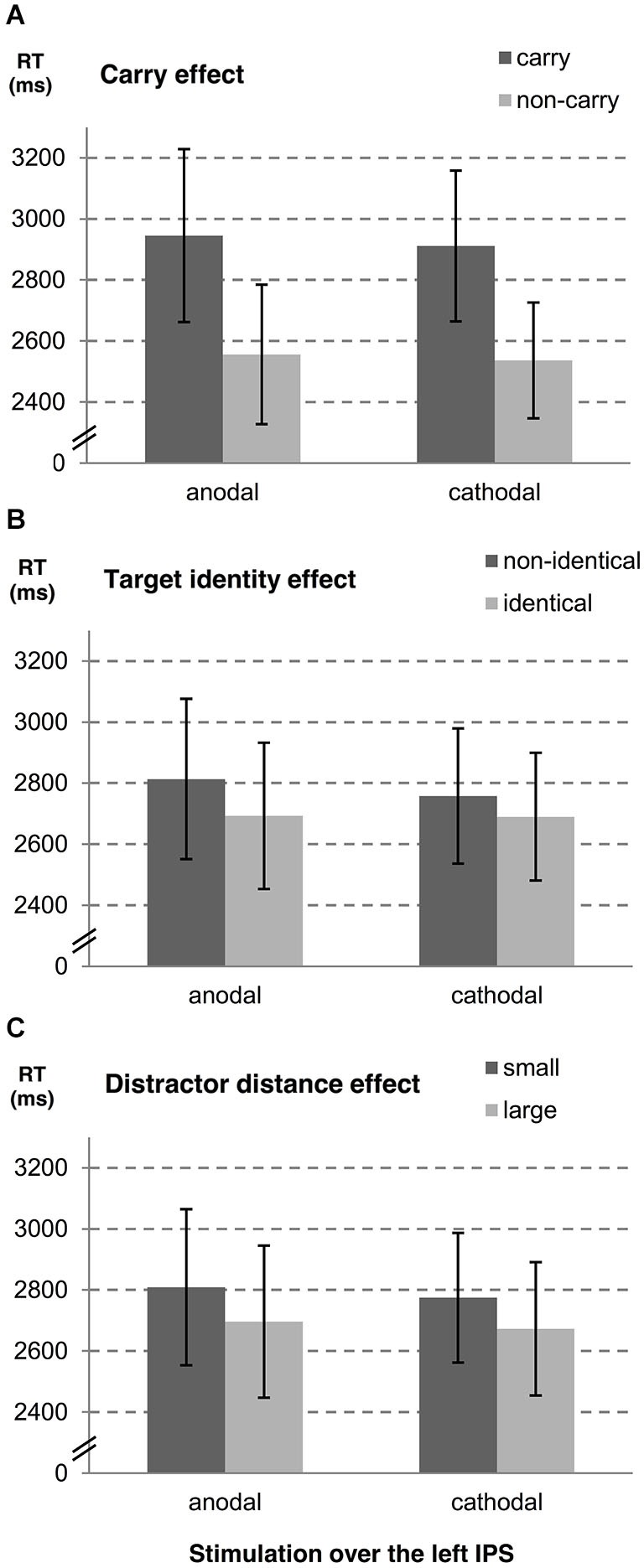
**Influences of tDCS over the left IPS on arithmetic performance**. The stimulation effects for the left hemisphere are shown for **(A)** carry-over, **(B)** target identity and **(C)** distractor distance. Error bars indicate the coincidence intervals.

In a second step, we examined influences of stimulation type on the carry effect, distractor distance effect, and the target identity effect for left and right hemispheres, separately. A summary of estimates, their *SE*, and the respective *t*- and *p*-values is given in Table 1 (see also Figures 2, 3). The analyses revealed reliable main effects of carry-over, distractor distance and target identity indicating that responses were faster when (i) the problem did not require a carry-over, (ii) distractor distance was large, and (iii) the target was the correct result of the problem. Additionally, we found a significant interaction between carry and stimulation type for the right hemisphere indicating a larger carry effect for anodal than for cathodal stimulation (see Figure 2A).

**Table 1:** **Estimates (in ms), *SE*, their respective *t*- and *p*-values of fixed effects and the standardized effect sizes (*ES*) in the addition task separately for left and right stimulation side**.

Effect	Estimate	*SE*	*df*	***t***-value	*p*	*ES*
Right side
Carry-over	367.88	41.27	27.14	8.91	<0.001	0.42
Stimulation type × carry-over	72.66	40.39	23.48	1.80	0.042	0.08
Target identity	108.96	20.40	28.54	5.34	<0.001	0.12
Stimulation type × target identity	1.21	32.70	23.34	0.04	0.485	<0.01
Distractor distance	105.56	23.94	30.77	4.41	<0.001	0.12
Stimulation type × distractor distance	−10.20	28.13	134.17	−0.36	0.641	−0.01
Left side
Carry-over	381.52	44.35	26.73	8.60	<0.001	0.44
Stimulation type × carry-over	14.14	28.46	118.03	0.50	0.310	0.02
Target identity	93.90	20.96	29.35	4.48	<0.001	0.11
Stimulation type × target identity	52.84	35.70	23.90	1.48	0.076	0.06
Distractor distance	107.38	22.21	33.00	4.83	<0.001	0.12
Stimulation type × distractor distance	11.65	31.76	42.87	0.37	0.358	0.01

*Note: p-values for one-tailed tests of our specific hypotheses*.

### Color word stroop task

For the control stroop task, we first ran a LMM incorporating the factor stimulation. Comparable to the addition task, the main effect of stimulation was not significant indicating that RT did not differ between stimulation conditions (right anodal: 655 ms, left anodal: 646 ms, sham: 652 ms, right cathodal: 638 ms, left cathodal: 648 ms; *F*_(4,18.74)_ = 0.42, *p* = 0.791).

Afterwards, the LMMs including stimulation type and congruency and their interaction as fixed effects for left and right hemisphere were conducted. The analysis revealed a significant main effect of congruency indicating faster responses for congruent than incongruent trials for the stimulation of both hemispheres (right side: 605 ms vs. 660 ms, respectively; estimate = 54.91, *SE* = 12.96, *t*_(19.06)_ = 4.24, *p* < 0.001, *ES* = 0.32; left side: 605 ms vs. 663 ms, respectively; estimate = 58.09 ms, *SE* = 10.92 ms, *t*_(21.45)_ = 5.32, *p* < 0.001, *ES* = 0.31). Interactions between stimulation type and congruency were not significant for the left and right hemisphere (both *t* < 0.70, *p* > 0.500, *ES* < 0.03).

## Discussion

In this study, the influence of bi-cephalic unilateral tDCS over the right or left IPS on the effects of carry-over, target identity, and distractor distance in mental addition was investigated to evaluate the casual structure-function relationship between the IPS and numerical processing separately for both hemispheres. Results indicated tDCS over the right IPS to moderate the carry effect: the carry effect was reliably larger under anodal than cathodal stimulation. On the other hand, tDCS did not influence the effect of target identity significantly. Moreover, corroborating our hypothesis the effect of distractor distance was not affected by unilateral tDCS. This is in line with the findings of Klein et al. (2013) suggesting that number magnitude information is processed in the IPS bilaterally, so that bilateral tDCS is needed to affect the distractor distance effect. Finally, it is important to note that these effects of unilateral tDCS were specific to number processing because we did not observe any stimulation effects on the stroop task controlling for unspecific tDCS effects. In the following the effects of tDCS on mental arithmetic will be discussed in turn.

### Carry-over and the right intraparietal cortex

The current data indicate that the carry effect is modulated by tDCS over the right IPS, with the effect being increased by anodal compared to cathodal stimulation. Considering the carry effect to reflect processes of place-value integration (Klein et al., 2010b; see Nuerk et al., in press for a detailed discussion), the observation of this lateralization in the right hemisphere is in line with recent neuro-scientific evidence. In an fMRI study Göbel et al. (2004) observed differential patterns of activation for the processing of single- and two-digit numbers within the right IPS. This first evidence for the specific processing of place-value information in the right IPS was further corroborated by the results of Wood et al. (2006) evaluating the neural correlates of two-digit number magnitude comparison. The authors found that the integration of separate and automatically activated representations of tens and units into the place-value structure of the Arabic number system was specifically associated with activation of the right anterior IPS. Interestingly, Cohen Kadosh et al. (2010) observed that the automatic activation of digit magnitude is also associated with the right hemisphere. Using contralateral reverse tDCS the authors found the size congruity effect in a numerical stroop task to be increased by anodal and decreased by cathodal tDCS of the right parietal lobe.

Taken together, our findings indicate that anodal tDCS over the right IPS may have enhanced automatic digit magnitude activation for tens and units separately. In turn, this may have made place-value integration—as required for the carry operation—more demanding which then caused the more pronounced carry effect in this stimulation condition. On the other hand, cathodal tDCS over the right IPS may have led to decreased automatic digit magnitude activation which in turn facilitated processes of place-value integration resulting in the smaller carry effect observed.

### Target identity and the intraparietal cortex

In contrast to our expectation we did not observe a significant modulation of the target identity effect by unilateral tDCS. We hypothesized that the effect of target identity may be associated with processes of familiarity recognition (i.e., recognizing the computed results within the solution probes presented) as also involved in arithmetic fact retrieval (e.g., Klein et al., 2013). These were suggested to be subserved—amongst other regions—by inferior parietal cortical sites including the angular gyrus (e.g., Dehaene et al., 2003) and should thus have been affected by our stimulation. However, the fact that the target identity effect remained unaffected by unilateral tDCS is in line with the assumption that we have stimulated only parts of the cortical network associated with familiarity recognition. Apart from the angular gyrus Klein et al. (2013) also observed the retrosplenial cortex (see also Shah et al., 2001; Klein et al., 2010b; see Vann et al., 2009 for a review) as well as the hippocampus (e.g., Montaldi and Mayes, 2010) to be involved in familiarity processing. Therefore, eventual interference due to tDCS on inferior parietal cortical sites may not have been sufficient to influence familiarity processing within the context of numerical cognition.

### Distractor distance and the bilateral intraparietal cortex

Importantly, the distractor distance effect was not modulated by unilateral tDCS of either the left or right IPS in the current study. This result adds to the finding of the study by Klein et al. (2013) showing that the effect of distractor distance was decreased by anodal and increased by cathodal bilateral tDCS over the IPS. Therefore, the data of the current study further support the proposition that number magnitude processing is subserved by the IPS bilaterally following a causal structure-function relationship (Pinel et al., 2001; Dehaene et al., 2003) by complementing the data for bilateral tDCS with the investigation of possible unilateral dissociations.

## Conclusion

Taken together, the current study evaluated the lateralization of specific numerical processes applying unilateral tDCS over the IPS. On the one hand, we observed converging evidence for number magnitude processing in the IPS bilaterally as it was not modulated by unilateral tDCS. On the other hand, we found indications for a right lateralization of place-value processing in line with recent neuro-scientific evidence and theoretical consideration. Finally, these stimulation effects were specific to number processing because more general cognitive processes were not affected.

In summary, these data indicate that the neuro-cognitive underpinnings of number processing may be more complex than initially assumed by the Triple Code Model (see Dehaene et al., 2003). In particular, unilateral influences have to be taken into account and the functional necessity of the cortical areas proposed needs to be evaluated more thoroughly. Besides lesion studies experiments using brain stimulation techniques such as tDCS but also TMS or transcranial random noise stimulation (tRNS; Snowball et al., 2013) might help to systematically evaluate causal function-structure relationships in number processing.

## Conflict of interest statement

The authors declare that the research was conducted in the absence of any commercial or financial relationships that could be construed as a potential conflict of interest.
